# Dietary Adequacy of Vitamin D and Calcium among Inuit and Inuvialuit Women of Child-Bearing Age in Arctic Canada: A Growing Concern

**DOI:** 10.1371/journal.pone.0078987

**Published:** 2013-11-04

**Authors:** Fariba Kolahdooz, Alison Barr, Cindy Roache, Tony Sheehy, Andre Corriveau, Sangita Sharma

**Affiliations:** 1 Aboriginal and Global Health Research Group, Department of Medicine, University of Alberta, Edmonton, Alberta, Canada; 2 Department of Food and Nutritional Sciences, University College Cork, Cork, Republic of Ireland; 3 Department of Health and Social Services, Government of the Northwest Territories, Yellowknife, Northwest Territories, Canada; Brigham & Women's Hospital, and Harvard Medical School, United States of America

## Abstract

**Background:**

Arctic populations are at an increased risk of vitamin D inadequacy due to geographic latitude and a nutrition transition. This study aimed to assess the adequacy of dietary vitamin D and calcium among women of child-bearing age in Arctic Canada.

**Methods:**

This study collected data from 203 randomly selected women of child-bearing age (19-44 years) in Nunavut and the Northwest Territories of Arctic Canada. Cross-sectional surveys using a validated quantitative food frequency questionnaire were analysed to determine the dietary adequacy of vitamin D and calcium and summarize the top foods contributing to vitamin D and calcium intake among traditional food eaters (TFE) and non-traditional food eaters (NTFE).

**Results:**

The response rate was between 69-93% depending on the community sampled. Mean BMIs for both TFE and NTFE were above the normal range. Traditional food eaters had a significantly higher median vitamin D intake compared with non-traditional eaters (TFE = 5.13±5.34 µg/day; NTFE = 3.5±3.22 µg/day, p = 0·004). The majority of women (87%) were below the Estimated Average Requirements (EAR) for vitamin D. Despite adequate median daily calcium intake in both TFE (1299±798 mg/day) and NTFE (992±704 mg/day; p = 0.0005), 27% of the study population fell below the EAR for calcium. Dairy products contributed the most to intake of vitamin D (TFE = 30.7%; NTFE = 39.1%) and calcium (TFE = 25.5%; NTFE = 34.5%).

**Conclusions:**

Inadequate dietary vitamin D intake is evident among Inuit and Inuvialuit women of child-bearing age in Arctic Canada. Promotion of nutrient-rich sources of traditional foods, supplementation protocols and/or expanded food fortification should be considered to address this nutrition concern.

## Introduction

Vitamin D and calcium insufficiency are increasingly recognized throughout the world, and those with limited solar ultraviolet B (UVB) exposure, dark skin pigmentation, the obese, elderly, pregnant women and breastfed infants are at high risk of vitamin D inadequacy [1]–[3]. Low vitamin D status has been associated with a list of diseases both skeletal and non-skeletal in nature, including rickets, osteoporosis, cancer, type 1 and type 2 diabetes, cardiovascular disease, multiple sclerosis, rheumatoid arthritis, inflammatory bowel disease, asthma, and schizophrenia [4]–[6]. Low calcium status has also been associated with some chronic diseases and oral health [7], and calcium supplementation is associated with a significant protective benefit in the prevention of pre-eclampsia and increased birth weight [8]. Therefore, there is growing concern that poor pre- and postnatal vitamin D and calcium status among women greatly increases an infant's susceptibility to these diseases [9]; [10].

The main source of vitamin D synthesized in the body is from ultraviolet B exposure; however, this is affected by factors such as time and duration of exposure, latitude, air pollution, extensive body coverage, sunscreen use and skin pigmentation [1]. Residents of Arctic nations have limited sun exposure and synthesis of vitamin D [1], hence those individuals may need dietary and supplemental sources of vitamin D for adequate nutrition [3]; [11]; [12]. Furthermore, Aboriginal Arctic populations have experienced a transition in diet and lifestyle over the past fifty years, resulting in decreased consumption of traditional foods, including fish, marine mammals, and organ meats, which are rich sources of vitamin D and calcium [13].

To date, few studies have reported dietary intake of vitamin D and calcium among women of child-bearing age in Arctic Canada. In Inuvik, Northwest Territories (NWT), mean daily intake of vitamin D was found to be significantly lower in native women compared to Caucasians [14]. A study of Inuit and Inuvialuit populations living in the Arctic also found that mean vitamin D intake was less than a quarter of the recommendation [15]; [16]. Furthermore, a review has revealed that the Canadian Aboriginal diet is low in calcium and vitamin D [17]. Another study of thirty-six Arctic communities found that in women, food insecurity was associated with significantly lower intake of calcium and vitamin D [18].

Conversely, the First Nations Bone Health Study reported that Canadian Aboriginal and Caucasian women in Manitoba met the Adequate Intake (AI) level for vitamin D, but rural Aboriginal women had lower vitamin D intake than urban Caucasian or Aboriginal women, with 32% of rural Aboriginal women being vitamin D deficient (serum 25 hydroxycholecalciferol (25 OH(D)) <37.5 nmol/l). The study also reported that rural Aboriginal women had lower calcium intake than urban Caucasian or Aboriginal women and only a minority of Aboriginal women met the AI level for calcium [14]. Another Canadian study of young Aboriginal women found that average intake of vitamin D met recommended levels, but calcium did not [19]. This study aimed to examine pooled data from culturally-appropriate quantitative food frequency questionnaires (QFFQs) collected twice over a period of approximately two years to determine dietary adequacy and the main food sources of vitamin D and calcium among Inuit and Inuvialuit women of child-bearing age.

## Materials and Methods

### Ethics statement

Ethical approval was obtained from the Committee on Human Studies at the University of Hawaii and the Office of Human Research Ethics at the University of North Carolina at Chapel Hill. The Nunavut Research Institute, as well as the Aurora Research Institute in Inuvik, approved and issued a research license for the study.

### Participant recruitment and data collection

All data were collected at baseline, 2008 to 2009, (prior to implementing the intervention) for the Healthy Foods North nutrition and lifestyle intervention. The setting, recruitment methods and data collection procedures have been described in detail elsewhere [12]; [15]. From a total of 34 communities in the NWT and 26 Nunavut communities, six communities were selected through communications with the Department of Health and Social Services in the Government of Nunavut and Government of NWT. Homes were randomly selected in the six communities in Nunavut and the NWT (three communities in each territory), using local housing maps. Participants were women aged 19–44 years, had lived in the community for at least six months, and were the main food preparers and shoppers for the household. Pregnant and breastfeeding women were excluded due to their different nutritional requirements. The communities in Nunavut have populations ranging from 800 to 1,500 people, each with two food stores. The communities in the NWT range in size from 400 to 3,500 people, with two food stores in smaller communities and one to three food stores in each community. The stores' food supplies are provided by airfreight year round and by barge/sealift during a small window of time in the summer months when the ice melts. In each territory, the largest communities represent regional administrative centres, while the smaller communities are comparatively more remote, and traditional activities, such as hunting and fishing, remain a part of daily life.

Trained field workers collected dietary data using a culturally appropriate, validated QFFQ developed specifically for the study population [20]. All questionnaires were interviewer administered. Participants were contacted up to seven times; if still unavailable, the interviewers moved on to the next household. Participants were informed about the objectives of the study after which they were asked to sign a consent form prior to the start of the interview. An interviewer fluent in the local language or an interpreter was used for participants whose primary language was not English. Participants were asked to report the frequency of consumption over a 30-day period by choosing from eight categories, which ranged from ‘never’ to ‘2≥ times/day’. Three-dimensional food models (NASCO Company, Fort Atkinson, Wisconsin 53538), household units (e.g. bowls, mugs, and spoons) or standard units (e.g. teaspoon) and local food packages were carefully chosen with input from local communities to best estimate the weight per portion of foods and beverages consumed. Supplement information was collected as part of the QFFQ. All dietary data from the QFFQs were coded and analyzed using Nutribase Clinical Nutrition Manager Version 9 (Cybersoft Inc., Phoenix, AZ, USA), a computerized dietary database. Information on demographics and socioeconomic status were collected. Height and weight measurements were measured in duplicate by trained staff. Height was recorded to the nearest centimeter using an audiometer; a digital scale was used to measure weight to the nearest 500 g.

All data were examined and quality controlled and if any set of data was incomplete the interviewer contacted the respondent in order to obtain the missing information. Height and weight measurements were refused by two percent of participants, and therefore, self-reported values were used. The response rate was between 69–93% depending on the community sampled [21]. Participants were given a CAD $25 gift certificate for a local store to thank them for their time.

Descriptive statistics included body mass index (BMI) (normal weight <25 kg/m^2^, overweight 25–29.9 kg/m^2^, and obese ≥30 kg/m^2^), smoking status (yes/no), marital status (yes/no), education level (none or some junior high school/ junior high school or high school completed/ college, trade school or university completed) and Material Style of Life (MSL) score (≤7, 8–12, and >12). The MSL score was determined from an additive scale developed for use as a proxy for socioeconomic status among Aboriginal groups [22]. The mean portion size, frequency of consumption and total daily intake were computed for 150 individual foods and twenty-two food groups. The foods and food groups are shown in Appendix S1. Traditional eaters were defined, as those who consumed ≥300 g of traditional foods per day and non-traditional eaters were those consuming <300 g of traditional foods per day, based on the median intake of traditional foods among participants. Traditional meats/poultry include caribou, seal, musk ox, goose, and ptarmigan. Traditional fish include Arctic char and trout.

Average daily intakes of vitamin D and calcium among women of child-bearing age in Nunavut and the NWT were pooled from each territory. Average daily intakes of vitamin D and calcium were compared with the Recommended Dietary Allowances (RDAs) (15 µg/day for vitamin D and 1000 mg/day for calcium) for women aged 19–30 years and 31–44 years. Dietary adequacy of vitamin D and calcium was determined using their respective Estimated Average Requirements (EAR) (10 µg/day for vitamin D and 800 mg/day for calcium) [23]. The top ten foods contributing to vitamin D and calcium intake were also determined.

A nonparametric Wilcoxon rank-sum test was performed to test for differences in intakes of vitamin D and calcium between traditional and non-traditional food eaters. Differences were considered statistically significant at *p*<0.05. All statistical analyses were performed using SAS statistical software, version 9.2 (Cary, NC. SAS Institute Inc).

## Results

Data were collected from 205 Inuit and Inuvialuit women 19–44 years of age from six communities in Nunavut and the NWT. Demographic and socioeconomic characteristics of this study population are presented in Table 1. Two subjects whose estimated caloric intakes were high (>5000 kcal/d) were excluded, leaving 203 participants for the final analyses. The mean (and standard deviation) age of traditional food eaters (TFE) was higher than that of non-traditional food eaters (NTFE) (TFE = 35.3±5.9 year; NTFE = 33.3±7.5; p = 0.02). Mean BMIs were similar for both groups and higher than the normal range (TFE = 29.8±9.3; NTFE = 29.2±7.5 kg/m2; p = 0.6). Approximately 47% of TFE were obese compared with 53% of NTFE. Overall 80% of women smoked. Socioeconomic status, as measured by MSL score, did not vary significantly between TFE and NTFE. The majority of women in both groups reported being married or were in a common-law relationship. In both groups, more women completed junior high school or high school (TFE = 41%; NTFE = 53%) than women who had no formal education or completed some junior high school (TFE = 35%; NTFE = 25%) and women who had a partial or full college education (TFE = 23%; NTFE = 22%) (Table 1).

**Table 1:** Demographic and socioeconomic characteristics of traditional food eaters and non-traditional food eaters^a^ among Inuit and Inuvialuit women of child-bearing age (19-44 years) in Nunavut and the Northwest Territories.

Characteristics	TFE (n = 99)	NTFE (n = 104)	p-value
	Mean ± SD	Mean ± SD	
**Age (years)**	35.3±5.9	33.3±7.5	0.02^b^
**BMI (kg/m^2^)**	29.8±9.3	29.2±7.5	0.6^b^
	***n*** ** (%)** c	***n*** ** (%)** c	
**BMI (kg/m^2^)** e			
>30.0 (obese)	17 (20)	12 (16)	
25–29.9 (overweight)	28 (33)	24 (32)	
<25.0 (normal)	40 (47)	40 (53)	0.38^d^
**Smoking Status**			
Yes	77 (80)	80 (78)	
No	19 (20)	22 (22)	0.69^d^
**Material Style of Life score** f			
≤7	38 (41)	35 (39)	
8 – 12	24 (26)	28 (32)	
>12	30 (33)	26 (29)	0.89^d^
**Marital Status**			
Single	28 (29)	40 (39)	
Married or Common-law	67 (69)	61 (60)	0.38^d^
**Education Level**			
None – some junior HS	34 (35)	26 (25)	
Junior HS completed – HS completed	40 (41)	54 (53)	
College/trade school/university completed	22 (23)	22 (22)	0.18^d^

BMI, body mass index; HS, high school; NTFE, non-traditional food eaters; SD, standard deviation; TFE, traditional food eaters.

a Traditional eaters consumed >300 g and non-traditional eaters consumed ≤300 g of traditional foods/day.

b A Student t-test was performed.

c Numbers do not add up to the total *n* as a result of missing responses (BMI, n = 42; smoking status, n = 5; MSL score, n = 22; marital status, n = 7; educational level, n = 5).

d A Chi-square test was performed.

e 14 women from TFE and 28 women from NTFE refused to be measured for height and weight and also did not self report.

f Material Style of Life score was considered a proxy for socioeconomic status.

Overall, median vitamin D intake was 4.21±4.49 µg/day, which is lower than the RDA. Traditional food eaters had a significantly higher median vitamin D intake compared with NTFE (TFE = 5.13±5.34; NTFE = 3.5±3.22 µg/day, p = 0.004). The majority of women (87%) were below the EAR for vitamin D; a higher proportion of NTFEs ate less than the recommendation and were below the EAR (TFE = 82%; NTFE = 91%) (Table 2). Twenty-one percent of participants reported using one or more nutrient supplements in the past 30 days, with multivitamins being the most common. Three percent indicated taking a vitamin D-containing supplement. Despite adequate median daily calcium intake (1154±877 mg/day), 27% of women did not meet their EAR for calcium. The median intake of calcium was significantly higher in TFE (1299±798 mg/day) than NTFE (992±704 mg/day; p = 0.0005) and more NTFE (35%) than TFE (18%) were below the EAR for calcium (Table 2). About 5.1% of TFE and 14.4% of NTFE consumed <70% EAR, and 11.1% of TFE and 16.4% of NTFE consumed 70–90% EAR for calcium. We repeated the analyses by excluding the participants that refused to be measured for height and weight and also did not self report; the findings on vitamin D and calcium were similar between participants with and without BMI data (results not shown).

**Table 2:** Vitamin D and calcium intake and percent below the Estimated Average Requirements of traditional food eaters and non-traditional food eaters a among Inuit and Inuvialuit women of child-bearing age (19–44 years) in Nunavut and the Northwest Territories.

	TFE (n = 99)	NTFE (n = 104)	Total (n = 203)	p-value^b^
**Vitamin D (µg/day)** c				
**Mean** ± **SD**	7.11±7.47	4.88±4.72	5.97±6.30	0.02
**Median (**P25-P75**)**	5.13 (3.05–8.39)	3.50 (2.42–5.63)	4.21 (2.62–7.11)	
**n (%) below EAR** d	81 (82.0)	95 (91)	176 (87.0)	
**Calcium (mg/day)** c				
**Mean** ± **SD**	1428.0±649.0	1120.0±565.0	1270.0±625.0	0.0005
**Median (**P25–P75**)**	1299.0 (1002.4–1800.7)	992.0 (688.9–1393.0)	1154.0 (764.6–1641.2)	
**n (%) below EAR** d	18 (18.0)	36 (35.0)	54 (27.0)	

EAR, estimated average requirement; P25–P75, 25 percentile and 75 percentile; NTFE, non-traditional food eaters; SD, standard deviation; TFE, traditional food eaters.

a Traditional eaters consumed >300 g and non-traditional eaters consumed ≤300 g of traditional foods/day.

b Non-parametric Wilcoxon rank-sum test was performed to compare differences in intake between TFE and NTFE.

c The individual intake goal for vitamin D and calcium is referred to as the Recommended Dietary Allowance level. For Vitamin D and calcium the recommended intakes are15 µg/day and 1,000 mg/day respectively for women aged 19–50 years.

d Adequacy was determined using the Estimated Average Requirements levels of 10 µg/day vitamin D and 800 mg/day for calcium for women aged 19-50 years.

Dairy products (milk, yogurt, cheese and including eggs) topped the list contributing 30.7% and 39.1% to vitamin D intake amongst TFE and NTFE, respectively. Among TFE, traditional sea foods (such as muktuk, Arctic char, and trout) were the second highest contributor (20.5%), followed by non-nutrient-dense foods (NNDFs) (18.2%). The NNDFs group included butter/margarine, ice cream, chips, popcorn, crackers, pop, juice, candy, desserts as well as unsweetened and sweetened drinks. Among NTFE, NNDFs were the second highest contributor (20.0%). Pork and beef contributed approximately 13%, as the fourth and third contributors among TFE and NTFE, respectively. The remaining food groups on the list such as traditional land and sky foods each contributed less than 10% to vitamin D intake (Table 3). In total, traditional foods contributed over 30% of total vitamin D intake among TFE and less than 15% of total vitamin D intake among NTFE (Table 3).

**Table 3:** Top ten food groups contributing to vitamin D and calcium intake among traditional food eaters and non-traditional food eaters^a^ for Inuit and Inuvialuit women of child-bearing age in Nunavut and the Northwest Territories.

Traditional eaters		Non-traditional eaters	
***Food group***	***%(*** **µg) ** ***contribution to vitamin D***	***Food group***	***%(*** **µg) ** ***contribution to vitamin D***
**Dairy products and eggs**	30.7 (2.18)	**Dairy products and eggs**	39.1 (1.91)
**Traditional sea foods** b	20.5 (1.46)	**Non-nutrient-dense foods** c	20.0 (0.98)
**Non-nutrient-dense foods** c	18.2 (1.29)	**Beef & pork**	12.9 (0.63)
**Beef & pork**	13.1 (0.93)	**Traditional sea foods** b	11.3 (0.55)
**Traditional land foods** d	8.24 (0.59)	**Other foods** e	3.6 (0.18)
**Traditional sky foods** f	3.1 (0.22)	**Traditional sky foods** f	2.6 (0.13)
**Poultry**	1.4 (0.10)	**Nontraditional sea foods**	2.2 (0.11)
**White breads**	1.4 (0.10)	**Poultry**	2.1 (0.10)
**Potatoes**	1.3 (0.09)	**Potatoes**	2.0 (0.10)
**Soup**	0.8 (0.06)	**Traditional land foods** d	1.7 (0.08)
**Total**	**96.6 (6.87)**	**Total**	**97.5 (4.75)**
***Food group***	***%(mg) contribution to calcium***	***Food group***	***%(mg) contribution to calcium***
**Dairy products and eggs**	25.5 (364.1)	**Dairy products and eggs**	34.5 (386.4)
**Grain (breads)**	24.9 (355.6)	**Non-nutrient-dense foods** c	26.1 (292.3)
**Non-nutrient-dense foods** c	23.1 (329.9)	**Grain (breads)**	18.2 (203.8)
**Grain (noodles)**	4.3 (61.4)	**Other foods** e	5.12 (57.3)
**Traditional sea foods** b	4.0 (57.1)	**Grain (noodles)**	3.1 (34.7)
**Traditional land foods** d	3.8 (54.3)	**Beef & pork**	2.8 (31.4)
**Other foods** e	3.0 (42.8)	**Grain (cereals)**	1.5 (16.8)
**Beef & pork**	2.9 (41.4)	**Vegetables**	1.4 (15.9)
**Vegetables**	1.6 (22.8)	**Potatoes**	1.3 (14.6)
**Fresh fruit**	1.5 (21.4)	**Fresh fruit**	1.3 (14.6)
**Total**	**94.5 (1349.5)**	**Total**	**95.2 (1066.2)**

a Traditional eaters consumed >300 g and non-traditional eaters consumed ≤300 g of traditional foods/day.

b Includes char, trout, white fish, fish battered or fried, fish eggs, whale fat or oil.

c Includes sugar, hash browns, fried potato, French fries, salad dressing, pizza, ice cream, cake, pie, sweet donuts, Danish roll, pastries, potato chips, party mix, popcorn, crackers, wheat thins, sesame snacks, pilot biscuits, cookies, candy, chocolate, jelly, butter or margarine, sweetened drink, tang, juice, pop, energy drinks.

d Includes caribou, muskox, moose (boiled, baked, roast, dried, fried, burger, stir-fried with vegetables), meat organs, polar bear, rabbit or musk rat, caribou fat, Eskimo ice cream, caribou soup, stew, blood soup.

e Includes artificial sweetener, low fat or light salad dressing, popcorn, nuts, low fat spreads, low fat butter and margarines, peanut butter, unsweetened drinks, fruit juice, pop, diet cola, diet energy drinks.

f Includes wild birds, duck, ptarmigan, geese, swan and crane.

The main contributors to calcium intake were dairy products, (TFE = 25.5%; NTFE = 34.5%) followed by grains and NNDFs; overall, these foods contributed 77.8% and 83.4% to the total calcium intake amongst TFE and NTFE, respectively. Among TFE, traditional sea foods combined with traditional land foods contributed 7.8% of the total calcium intake. The contributions of beef and pork, vegetables, and fresh fruit to calcium intake were similar amongst TFE and NTFE (Table 3).

## Discussion

Dietary data on Inuit and Inuvialuit women of child-bearing age in the Canadian Arctic are limited; therefore, the present study is an important contribution to the current literature on dietary adequacy of vitamin D and calcium amongst this population. We found that the majority of women of child-bearing age consumed less than the recommended amount of vitamin D. Vitamin D cannot be produced cutaneously during several winter months due to decreased intensity of UVB radiation at latitudes 42° N and higher. Even during summer months, those living at high latitudes, particularly >60° N, require much longer UVB exposure time to produce cutaneous vitamin D [24]. If a woman of child-bearing age living in the Canadian Arctic becomes pregnant, her vitamin D needs increase and therefore must be met via dietary intake (food and/or supplements) [10]; [25]. This underscores the significant importance of adequate dietary vitamin D intake among women of child-bearing age in the Arctic.

Despite adequate median daily calcium intake, 27% of women did not meet their dietary daily recommendation for calcium. Amongst traditional food eaters 18% did not meet the calcium requirement, whilst 35% of women who were non-traditional eaters were below the EAR. Among traditional food eaters, traditional land, sea, and sky dishes contributed substantially to vitamin D intake, but not calcium. Among both TFE and NTFE, the contribution of grain to calcium intake was high, therefore resulting in calcium intakes similar to dairy products. This may also be related to the low intake of dairy products and high intake of grain in the Arctic.

The impacts of the nutrition transition are concerning because of the dietary inadequacies, including low vitamin D intake related to decreased traditional food and increased NNDF consumption, that have been documented in a number of studies [26]–[29]. Several traditional foods, such as fatty fish and sea mammals are rich sources of vitamin D; however, few other foods naturally contain vitamin D. Fortification of milk and margarine is required in Canada to help the population meet its needs [30]. Margarine was included in the NNDFs group in our analysis, which may explain why NNDFs were among the top 3rd greatest contributors to vitamin D intake as NNDFs are generally low in nutrients and high in energy, and fat [18]; [31]. High intake of NNDFs is associated with negative health consequences and is not recommended [28]. Our data support the importance of fortification of milk, as the dairy food group was the highest contributor to vitamin D and calcium intake. While traditional seafood was the second highest contributor, those who were NTFE had lower median daily intake of vitamin D than TFE. Overall the majority of the study populations did not meet vitamin D recommendations; however a greater percentage of non-traditional food eaters fell below the recommended intake for vitamin D. Vitamin D-rich traditional foods are seasonal (e.g. fatty fish); perhaps dietary vitamin D intake is greater among traditional eaters in some seasons, which was not captured with our data collection method.

Vitamin D deficiency, as measured by serum 25 OH(D) level, has been associated with obesity at various latitudes and has been implicated in susceptibility to the development of metabolic syndrome [4]; [32]. Although the relationship is unclear, it has been hypothesized that obesity could be a cause or a result of inadequate vitamin D and calcium status [33]. Smoking has also been associated with vitamin D inadequacy; among women in a Swiss population, current smokers consumed significantly less vitamin D and calcium than women who never smoked [34]. Smoking was also a significant determinant of summer- and wintertime 25 (OH)D levels in the general population of Estonia (latitude 59°N) [35]. Our data revealed a high prevalence of smoking. Further research is needed to determine if smoking and obesity are associated with vitamin D intake; however, these factors should be considered when assessing vitamin D status.

The adequacy of current dietary recommendations, especially for those at high latitudes, is hotly contested [36]. Vitamin D cannot be produced cutaneously during several winter months due to decreased intensity of UVB radiation at latitudes ≥42oN. Even during summer, those living at high latitudes, particularly >60oN, require much longer UVB exposure to synthesize vitamin D [24]. In Toronto, (latitude 43oN), low serum 25 (OH)D concentrations during half of the year (November-April) were observed despite self-reported adequate dietary vitamin D intake (200 IU/day) among women aged 18-35 years [37]. The authors concluded that adequate dietary intake, according to current recommendations, is not sufficient to prevent winter time hypovitaminosis D at this latitude. However, a study conducted in the UK (at 57oN) among post-menopausal women suggested that adequate dietary intake can attenuate seasonal variations of vitamin D status resulting from very low UVB [38].

The economic consequences of chronic disease are significant. Despite food fortification and dietary intake guidelines, vitamin D and calcium inadequacies are posing an increasing health threat to women of child-bearing age. Traditional foods were key sources of vitamin D in these population and nutrition programs aimed at improving dietary intake, including promotion of traditional foods, should be encouraged to combat nutrient inadequacies in the Arctic [12]. Vitamin D supplementation has been suggested for populations at high latitudes [30]; however, it will be important to consider if supplementation is an acceptable intervention among these populations, as supplement use is currently low [3]. Expanded food fortification in Canada has also been suggested to address low vitamin D intake [30]. The Institute of Medicine revised the RDA for vitamin D and calcium in collaboration with American and Canadian governments in 2010. The recommendation for vitamin D increased to 15 µg/day (600 IU) and for calcium increased to 1000 mg/day for women of child-bearing age. In acknowledging the descriptive nature of this work, it remains essential as a basis for future research. This may include exploration of how to remedy vitamin D inadequacy in Aboriginal women by providing evidence to revitalize traditional practices and revise vitamin D fortification and supplementation policies.

This study is not without limitations. All questionnaires were interviewer administered; therefore, response bias is possible. Additionally, a QFFQ may not be the most accurate tool to assess dietary adequacy; however, the QFFQs used in this study were validated in sub-studies and were found to accurately reflect the intake of these populations [20].

## Conclusions

Inadequate intake of vitamin D and calcium is a growing concern for the health of Inuit and Inuvialuit women of child-bearing age in Arctic Canada. Traditional foods were the main contributors to vitamin D and calcium intake among the Inuit, which is evident in the results of high intake of vitamin D among traditional food eaters. Therefore, the promotion of nutrient-rich sources of traditional foods should be considered to address vitamin D inadequacy among Arctic populations.

## Supporting Information

Appendix S1 **Food items that contributed to each food group.**
(DOC)
