# Supplementary material for: Dietary Adequacy of Vitamin D and Calcium among Inuit and Inuvialuit Women of Child-Bearing Age in Arctic Canada: A Growing Concern
Source: PLoS One. 2013 Nov 4;8(11):e78987. doi: 10.1371/journal.pone.0078987 (PMC3817094; doi:10.1371/journal.pone.0078987)
Supplement: Appendix S1 — Food items that contributed to each food group. (DOC) [file pone.0078987.s001.doc]

**Appendix S1. Food items that contributed to each food group**

| **Food groups** | **Individual food items on the quantitative food frequency questionnaire** |
| --- | --- |
| **Total fruit** | Apple, orange, banana, mango, grapes, strawberries, kiwi, peaches, nectarines, blueberries, raspberries, blackberries, fruit salad fresh, any canned fruit or fruit cocktail canned in syrup, frozen fruit, dried fruits and raisins |
| **Total vegetables** | Corn on the cob, corn, carrot, other fresh vegetables, salad in a bowl, tomatoes canned, vegetables canned, frozen |
| **Non-nutrient dense foods (NNDFs)** | Sugar in tea, coffee or cereal, hash browns or fried potato, french fries, salad dressing, pizza, ice cream, cake, pie, sweet donuts, Danish roll, pastries, potato chips, nacho chips, party mix, popcorn, crackers, wheat thins, sesame snacks, pilot biscuits, cookies, candy, chocolate, jelly, cereal bars, butter or margarine, sweetened drink, tang, juice, pop, energy drinks |
| **Dairy products and eggs** | Any kind of milk, yogurt, hard cheese or processed cheese, eggs any kind, chocolate milk, powder or liquid coffee creamer (regular or flavoured) |
| **Grain/ starch** |  |
| White Breads | Bannock, fried or baked, white bread, including toast, rolls, buns, pancakes or waffles |
| Whole wheat bread | whole wheat bread including toast, buns |
| Cereals | Sweet cereals, low sugar cereals, rice krispies, cheerios, mini wheat, harvest crunch, bran flakes oats, porridge |
| Noodles | Spaghetti or noodle without meat, macaroni |
| Rice | Rice, any kind |
| Potatoes | Potato salad, potato baked or boiled, mashed potato, hash browns and French fries |
| **Non-traditional meat** |  |
| Beef/pork | Beef steak, beef hamburgers, meat pie, sloppy joe, beef stir fried, spaghetti with ground beef/musk ox or beef ravioli, pork or beef ribs, pork chops, pork roast |
| Processed meats | Bologna/salami, Klik/canned meat, beef/musk ox jerky, pepperoni sticks, ham, hot dogs/wieners/sausages, bacon fried |
| Chicken/turkey | Chicken nuggets/popcorn chicken, chicken wings, chicken leg fried (KFC), chicken leg baked/boiled/roasted, chicken breast fried (KFC), chicken breast baked/boiled/roasted |
| **Traditional meats** |  |
| Traditional land meat | Caribou, musk ox, moose including boiled, baked, roast, dried, fried, burger, stir-fried with vegetables, or offal including liver, heart and kidney, polar bear, rabbit or musk rat, caribou fat, Eskimo ice cream, caribou soup, stew, blood soup |
| Traditional sea meat | Char (raw/smoked/boiled/dried), trout (raw/baked/boiled/dried), white fish raw/dried, fish battered or fried, fish eggs, whale fat or oil |
| Traditional sky meat | Wild birds including duck, ptarmigan, geese, swan and crane |
| **Other foods** | Artificial sweetener, salad dressing, low fat or light, popcorn, nuts, low fat spreads and low fat butter and margarines, peanut butter, unsweetened drinks, fruit juice, pop, diet cola, diet energy drinks |
| **Tea/coffee** | Tea and coffee any kind |
| **Alcohol** | Liquor including rum, whiskey, vodka or gin, beer or coolers, wine any kind |
